# Supplementary material for: Changes in serum amino acid levels in non-small cell lung cancer: a case-control study in Chinese population
Source: PeerJ. 2022 Apr 20;10:e13272. doi: 10.7717/peerj.13272 (PMC9034703; doi:10.7717/peerj.13272)
Supplement: Supplemental Information 1 [file peerj-10-13272-s001.docx]

**Table S1. Verification results of linearity.**

| Analytes | Linear range (nmol/L) | Linear equation | linear correlation coefficient (r^2^) |
| --- | --- | --- | --- |
| Gly | 10.0 - 400 | y=0.9889x+0.7899 | 0.9995 |
| Ser | 5.0 - 200 | y=1.0867x-4.8820 | 0.9956 |
| Pro | 10.0 - 400 | y=1.1750x-19.2948 | 0.9870 |
| Cys | 2.5 - 100 | y=1.0622x-1.9768 | 0.9986 |
| Asn | 2.5 - 100 | y=1.0122x-0.4112 | 0.9995 |
| Asp | 2.5 - 100 | y=0.9642x+0.9463 | 0.9952 |
| Leu | 5.0 - 200 | y=1.0660x-3.6368 | 0.9985 |
| Gln | 20.0 - 800 | y=1.0136x-4.1173 | 0.9993 |
| Glu | 2.5 - 100 | y=1.0351x-0.8931 | 0.9993 |
| Met | 2.5 - 100 | y=1.1754x-4.9241 | 0.9891 |
| His | 2.5 - 100 | y=0.9603x+0.7086 | 0.9993 |
| Phe | 2.5 - 100 | y=1.0156x-0.3253 | 0.9995 |
| Arg | 5.0 - 200 | y=0.8775x+6.3525 | 0.9905 |
| Cit | 2.5 - 100 | y=0.9842x+0.3703 | 0.9986 |
| Tyr | 2.5 - 100 | y=1.1083x-3.0716 | 0.9958 |
| Trp | 2.5 - 100 | y=1.0277x-0.9256 | 0.9996 |
| Orn | 2.5 - 100 | y=0.9075x+2.4572 | 0.9909 |
| Lys | 5.0 - 200 | y=0.9433x+2.9873 | 0.9924 |
| Ala | 20.0 - 800 | y=1.1784x-38.9806 | 0.9908 |
| Val | 10.0 - 400 | y=1.2015x-22.3155 | 0.9855 |
| Thr | 5.0 - 200 | y=1.0762x-4.0079 | 0.9983 |
| Ile | 2.5 - 100 | y=1.0156x-0.3994 | 0.9977 |

**Table S2. Verification results of precision.**

|  | Low level ^a^ | | | High level ^a^ | | |
| --- | --- | --- | --- | --- | --- | --- |
| Analytes | Target value (nmol/L) | Intra-  day (CV%) | Inter-  day (CV%) | Target value (nmol/L) | Intra-  day (CV%) | Inter-  day (CV%) |
| Gly | 10.0 | 7.23 | 13.10 | 200 | 7.93 | 12.60 |
| Ser | 5.0 | 7.62 | 20.20 | 100 | 12.60 | 13.40 |
| Pro | 10.0 | 5.67 | 13.00 | 200 | 1.30 | 5.50 |
| Cys | 2.5 | 3.15 | 9.77 | 50 | 3.05 | 12.80 |
| Asn | 2.5 | 3.10 | 11.30 | 50 | 3.94 | 11.10 |
| Asp | 2.5 | 9.94 | 19.60 | 50 | 6.32 | 18.40 |
| Leu | 5.0 | 5.40 | 8.44 | 100 | 3.07 | 4.37 |
| Gln | 20.0 | 5.14 | 14.60 | 400 | 4.00 | 14.90 |
| Glu | 2.5 | 6.63 | 8.05 | 50 | 6.30 | 9.91 |
| Met | 2.5 | 2.34 | 21.80 | 50 | 3.91 | 4.61 |
| His | 2.5 | 6.45 | 14.20 | 50 | 7.53 | 12.60 |
| Phe | 2.5 | 4.78 | 12.00 | 50 | 4.46 | 6.36 |
| Arg | 5.0 | 3.24 | 11.20 | 100 | 4.91 | 11.00 |
| Cit | 2.5 | 7.52 | 14.80 | 50 | 7.68 | 11.90 |
| Tyr | 2.5 | 5.33 | 22.20 | 50 | 5.38 | 7.89 |
| Trp | 2.5 | 2.45 | 23.10 | 50 | 4.83 | 6.17 |
| Orn | 2.5 | 3.46 | 14.10 | 50 | 8.55 | 9.68 |
| Lys | 5.0 | 9.11 | 14.40 | 100 | 8.78 | 18.00 |
| Ala | 20.0 | 8.30 | 14.80 | 400 | 4.06 | 7.50 |
| Val | 10.0 | 5.61 | 14.30 | 200 | 3.62 | 5.22 |
| Thr | 5.0 | 3.29 | 7.20 | 100 | 4.35 | 7.81 |
| Ile | 2.5 | 5.73 | 6.00 | 50 | 7.36 | 13.90 |

^a^ The quality controls were assayed five replicates on each day over five days.

**Table S3. Verification results of accuracy.**

| Analytes | Spiked  Concentration 1  (μ mol/L) | Recovery (%） | Spiked  Concentration 2  (μ mol/L) | Recovery 2 (%） |
| --- | --- | --- | --- | --- |
| Gly | 120 | 101.4 | 200 | 105.3 |
| Ser | 60 | 99.7 | 100 | 105.1 |
| Pro | 120 | 120.7 | 200 | 119.1 |
| Cys | 30 | 114.2 | 50 | 128.1 |
| Asn | 30 | 111.8 | 50 | 104.1 |
| Asp | 30 | 100.0 | 50 | 100.0 |
| Leu | 60 | 109.6 | 100 | 109.7 |
| Gln | 240 | 104.3 | 400 | 98.2 |
| Glu | 30 | 100.4 | 50 | 101.3 |
| Met | 30 | 115.0 | 50 | 110.8 |
| His | 30 | 128.3 | 50 | 118.3 |
| Phe | 30 | 103.9 | 50 | 102.3 |
| Arg | 60 | 94.3 | 100 | 102.2 |
| Cit | 30 | 82.1 | 50 | 84.5 |
| Tyr | 30 | 103.3 | 50 | 105.2 |
| Trp | 30 | 66.0 | 50 | 81.7 |
| Orn | 30 | 110.7 | 50 | 110.1 |
| Lys | 60 | 109.3 | 100 | 107.6 |
| Ala | 240 | 110.8 | 400 | 111.7 |
| Val | 120 | 110.3 | 200 | 103.7 |
| Thr | 60 | 106.6 | 100 | 100.3 |
| Ile | 30 | 109.8 | 50 | 110.1 |

**Table S4. Verification results of analytical sensitivity.**

| Analytes | LOQ (μ mol/L) ^a^ | LOD (μ mol/L) ^b^ |
| --- | --- | --- |
| Gly | 2.50 | 1.250 |
| Ser | 1.25 | 0.625 |
| Pro | 2.50 | 1.250 |
| Cys | 2.50 | 0.625 |
| Asn | 0.63 | 0.313 |
| Asp | 2.50 | 0.313 |
| Leu | 0.63 | 0.313 |
| Gln | 2.50 | 1.250 |
| Glu | 2.50 | 0.313 |
| Met | 2.50 | 0.313 |
| His | 0.31 | 0.156 |
| Phe | 2.50 | 0.312 |
| Arg | 0.63 | 0.313 |
| Cit | 0.63 | 0.313 |
| Tyr | 2.50 | 0.313 |
| Trp | 0.31 | 0.156 |
| Orn | 0.63 | 0.313 |
| Lys | 1.25 | 0.630 |
| Ala | 20.00 | 2.500 |
| Val | 10.00 | 1.250 |
| Thr | 5.00 | 0.630 |
| Ile | 0.31 | 0.156 |

^a^ The limit of quantification (LOQ) was defined as the analyte peak with a signal-to-noise of 10, and was established based on the criteria of maintaining the trueness within 80-120% and the variable coefficient (CV) <20%.

^b^ The limit of detection (LOD) was defined as the analyte peak with a signal-to-noise of 3.

**Table S5. Verification results of matrix effect.**

| Analytes | Set1 ^a^ | Set2 ^b^ | Set3 ^c^ | Set4 ^d^ | /Set3-Set4/／Ste4 (%) |
| --- | --- | --- | --- | --- | --- |
| Gly | 3.9E+06 | 2.2E+07 | 1.3E+07 | 1.3E+07 | 1.05 |
| Ser | 3.1E+06 | 6.6E+06 | 4.7E+06 | 4.8E+06 | 3.10 |
| Pro | 4.5E+07 | 1.8E+06 | 2.2E+07 | 2.3E+07 | 5.64 |
| Cys | 4.8E+05 | 5.6E+05 | 5.3E+05 | 5.2E+05 | 0.77 |
| Asn | 5.6E+06 | 8.6E+06 | 6.6E+06 | 7.1E+06 | 6.63 |
| Asp | 1.6E+06 | 1.7E+05 | 8.8E+05 | 8.8E+05 | 0.43 |
| Leu | 3.9E+07 | 9.2E+06 | 2.1E+07 | 2.4E+07 | 11.10 |
| Gln | 2.1E+07 | 2.0E+07 | 1.9E+07 | 2.0E+07 | 6.27 |
| Glu | 4.2E+06 | 1.0E+06 | 2.3E+06 | 2.6E+06 | 12.30 |
| Met | 8.2E+06 | 1.6E+06 | 4.4E+06 | 4.9E+06 | 9.01 |
| His | 5.5E+05 | 7.9E+06 | 4.4E+06 | 4.2E+06 | 3.07 |
| Phe | 1.7E+07 | 1.4E+07 | 1.4E+07 | 1.5E+07 | 8.13 |
| Arg | 1.9E+06 | 2.4E+06 | 2.0E+06 | 2.2E+06 | 5.68 |
| Cit | 1.0E+06 | 1.4E+05 | 5.6E+05 | 5.8E+05 | 3.67 |
| Tyr | 9.2E+06 | 1.5E+07 | 9.9E+06 | 1.2E+07 | 18.30 |
| Trp | 9.2E+06 | 1.1E+07 | 8.6E+06 | 1.0E+07 | 15.50 |
| Orn | 6.5E+05 | 1.2E+05 | 3.3E+05 | 3.9E+05 | 15.70 |
| Lys | 9.8E+05 | 1.3E+06 | 1.1E+06 | 1.1E+06 | 7.29 |
| Ala | 2.8E+07 | 1.6E+07 | 1.9E+07 | 2.2E+07 | 14.40 |
| Val | 4.6E+07 | 9.1E+06 | 2.7E+07 | 2.8E+07 | 1.91 |
| Thr | 8.4E+06 | 1.0E+07 | 8.1E+06 | 9.3E+06 | 12.80 |
| Ile | 2.2E+07 | 4.1E+06 | 1.2E+07 | 1.3E+07 | 10.90 |

^a^ The pure solution of the target analytes.

^b^ The serum sample.

^c^ Mixing Set1 and Set2 in a 1:1 mixture.

^d^ The mean peak area of the pure solution sample (Set1) and the serum sample (Set1).
