# Supplementary material for: Changes in serum amino acid levels in non-small cell lung cancer: a case-control study in Chinese population
Source: PeerJ. 2022 Apr 20;10:e13272. doi: 10.7717/peerj.13272 (PMC9034703; doi:10.7717/peerj.13272)
Supplement: Supplemental Information 2 [file peerj-10-13272-s002.docx]

**Table S6. Demographics and clinical characteristics based on histological types.**

|  |  | Adenocarcinoma | SCC | *P*-value |
| --- | --- | --- | --- | --- |
|  |  | N=133 | N=57 |  |
| Gender |  |  |  | <0.001 |
|  | Males | 77 (57.9%) | 51 (89.5%) |  |
|  | Females | 56 (42.1%) | 6 (10.5) |  |
| Age |  | 58 (34-81) | 58 (39-79) | 0.637 |
| Smoking ^a^ |  |  |  | <0.001 |
|  | Yes | 53 (39.8%) | 42 (73.7%) |  |
|  | No | 80 (60.2%) | 15 (26.3%) |  |
| Drinking ^a^ |  |  |  | 0.031 |
|  | Yes | 31 (23.3%) | 22 (38.6%) |  |
|  | No | 102 (76.7%) | 35 (61.4%) |  |
| BMI ^a^ |  |  |  | 0.077 |
|  | <18.5 | 12 (9.0%) | 10 (17.5%) |  |
|  | 18.5-24.0 | 65 (48.9%) | 33 (57.9%) |  |
|  | 24.0-28.0 | 48 (36.1%) | 13 (22.8%) |  |
|  | ≥28.0 | 8 (6.0%) | 1 (1.8%) |  |

Chi-square test was used to compare categorical variables. Chi-square test: (1) gender, χ^2^=18.100, df=1, (2) smoking, χ^2^=18.271, df=1, (3) drinking, χ^2^=4.637, df=1, (4) BMI, χ^2^=6.854, df=3.

T-test (normal distribution) was employed to compare continuous variables. T-test: (1) age, F=2.884, df=188, 95%CI (-3.113-1.910).
