# Supplementary material for: Changes in serum amino acid levels in non-small cell lung cancer: a case-control study in Chinese population
Source: PeerJ. 2022 Apr 20;10:e13272. doi: 10.7717/peerj.13272 (PMC9034703; doi:10.7717/peerj.13272)
Supplement: Supplemental Information 3 [file peerj-10-13272-s003.docx]

**Table S7. Demographics and clinical characteristics based on clinical stage.**

|  |  | Early-stage | Late-stage | *P*-value |
| --- | --- | --- | --- | --- |
|  |  | N=85 | N=105 |  |
| Gender |  |  |  | 0.004 |
|  | Males | 49 (57.6%) | 81 (77.1%) |  |
|  | Females | 36 (42.4%) | 24 (22.9%) |  |
| Age |  | 58 (36-81) | 58 (34-79) | 0.773 |
| Smoking ^a^ |  |  |  | 0.013 |
|  | Yes | 34 (40.0%) | 61 (58.1%) |  |
|  | No | 51 (60.0%) | 44 (41.9%) |  |
| Drinking ^a^ |  |  |  | 0.005 |
|  | Yes | 15 (17.6%) | 38 (36.2%) |  |
|  | No | 70 (82.4%) | 67 (63.8%) |  |
| BMI ^a^ |  |  |  | 0.407 |
|  | <18.5 | 8 (9.4%) | 10 (9.5%) |  |
|  | 18.5-24.0 | 43 (50.6%) | 57 (54.3%) |  |
|  | 24.0-28.0 | 32 (37.6%) | 31 (29.5%) |  |
|  | ≥28.0 | 2 (2.4%) | 7 (6.7%) |  |

Chi-square test was used to compare categorical variables. Chi-square test: (1) gender, χ^2^=χ^2^=8.263, df=1, (2) smoking, χ^2^=6.152, df=1, (3) drinking, χ^2^=8.031, df=1, (4) BMI, χ^2^=2.903, df=3.

T-test (normal distribution) was employed to compare continuous variables. T-test: (1) age, F=0.094, df=188, 95%CI (-1.943-2.610).
