# Supplementary material for: Changes in serum amino acid levels in non-small cell lung cancer: a case-control study in Chinese population
Source: PeerJ. 2022 Apr 20;10:e13272. doi: 10.7717/peerj.13272 (PMC9034703; doi:10.7717/peerj.13272)
Supplement: Supplemental Information 4 [file peerj-10-13272-s004.docx]

**Table S8. Metabolic pathway analysis.**

| Pathway name | Total | Hits | *P*-value | -log(*P*) | Holm adjust | FDR | Impact |
| --- | --- | --- | --- | --- | --- | --- | --- |
| Glycine, serine and threonine metabolism | 33 | Ser, Gly, Cys | 0.001 | 2.889 | 0.102 | 0.018 | 0.463 |
| Alanine, aspartate and glutamate metabolism | 28 | Glu, Asp, Asn | 0.001 | 3.102 | 0.064 | 0.017 | 0.421 |
| Arginine biosynthesis | 14 | Glu, Asp, Cit, Orn | <0.001 | 5.876 | <0.001 | <0.001 | 0.406 |

The MetaboAnalyst 5.0 was used for metabolic pathway analysis.
