# Supplementary material for: Changes in serum amino acid levels in non-small cell lung cancer: a case-control study in Chinese population
Source: PeerJ. 2022 Apr 20;10:e13272. doi: 10.7717/peerj.13272 (PMC9034703; doi:10.7717/peerj.13272)
Supplement: Supplemental Information 5 [file peerj-10-13272-s005.docx]

| Amino acid | Early-stage | | | | |  | Late-stage | | | | | Fold  Change ^e^ | *P*-value ^e^ | *P*-FDR ^e^ |
| --- | --- | --- | --- | --- | --- | --- | --- | --- | --- | --- | --- | --- | --- | --- |
|  | N=85 | | | | |  | N=103 | | | | |  |  |  |
|  | Median | IQR | Fold  Change ^c^ | *P*-value ^c^ | *P*-FDR ^c^ |  | Median | IQR | Fold  Change ^d^ | *P*-value ^d^ | *P*-FDR ^d^ |  |  |  |
| Phenylalanine ^a^ | 96.90 | 40.10 | 1.00 | 0.800 | 0.838 |  | 112.00 | 46.20 | 1.16 | <0.001 | **<0.001** | 1.16 | 0.004 | 0.088 |
| Tryptophan ^a^ | 51.90 | 26.70 | 0.90 | 0.055 | 0.152 |  | 51.10 | 23.90 | 0.89 | 0.024 | **0.048** | 0.98 | 0.929 | 0.929 |
| Isoleucine ^a^ | 84.50 | 43.70 | 1.03 | 0.168 | 0.264 |  | 88.40 | 36.30 | 1.08 | 0.021 | **0.048** | 1.05 | 0.582 | 0.800 |
| Leucine ^a^ | 170.00 | 66.00 | 1.06 | 0.434 | 0.617 |  | 173.00 | 60.00 | 1.07 | 0.027 | 0.050 | 1.02 | 0.341 | 0.757 |
| Methionine ^a^ | 27.80 | 11.90 | 0.94 | 0.496 | 0.633 |  | 27.70 | 11.40 | 0.93 | 0.714 | 0.827 | 1.00 | 0.784 | 0.862 |
| Lysine ^a^ | 237.00 | 120.00 | 1.07 | 0.158 | 0.264 |  | 222.00 | 116.50 | 1.00 | 0.810 | 0.849 | 0.94 | 0.283 | 0.757 |
| Valine ^a^ | 295.00 | 130.50 | 1.07 | 0.449 | 0.617 |  | 290.00 | 99.50 | 1.05 | 0.164 | 0.241 | 0.98 | 0.712 | 0.862 |
| Threonine ^a^ | 152.00 | 84.00 | 1.03 | 0.091 | 0.174 |  | 150.00 | 75.00 | 1.01 | 0.860 | 0.860 | 0.99 | 0.216 | 0.679 |
| Glycine ^b^ | 342.00 | 178.50 | 1.05 | 0.095 | 0.174 |  | 367.00 | 171.50 | 1.13 | 0.002 | **0.007** | 1.07 | 0.437 | 0.757 |
| Serine ^b^ | 185.00 | 88.50 | 1.08 | 0.069 | 0.152 |  | 191.00 | 72.50 | 1.12 | 0.003 | **0.009** | 1.03 | 0.462 | 0.757 |
| Asparagine ^b^ | 61.40 | 27.90 | 1.06 | 0.041 | 0.152 |  | 63.70 | 22.10 | 1.10 | 0.009 | **0.025** | 1.04 | 0.765 | 0.862 |
| Aspartic acid ^b^ | 40.40 | 32.50 | 1.24 | 0.001 | 0.022 |  | 50.30 | 34.20 | **1.55** | <0.001 | **<0.001** | 1.25 | 0.008 | 0.088 |
| Cysteine ^b^ | 64.50 | 45.20 | 1.20 | 0.032 | 0.152 |  | 72.70 | 54.20 | **1.35** | <0.001 | **<0.001** | 1.13 | 0.122 | 0.447 |
| Glutamic acid ^b^ | 119.00 | 70.00 | 1.14 | 0.004 | **0.044** |  | 136.00 | 82.00 | **1.30** | <0.001 | **<0.001** | 1.14 | 0.089 | 0.392 |
| Glutamine ^b^ | 541.00 | 256.00 | 1.07 | 0.043 | 0.152 |  | 516.00 | 194.50 | 1.02 | 0.333 | 0.458 | 0.95 | 0.391 | 0.757 |
| Histidine ^b^ | 66.20 | 32.50 | 1.10 | 0.068 | 0.152 |  | 58.30 | 31.20 | 0.97 | 0.461 | 0.597 | 0.88 | 0.026 | 0.191 |
| Proline ^b^ | 209.00 | 123.50 | 0.98 | 0.855 | 0.855 |  | 225.00 | 90.50 | 1.05 | 0.123 | 0.193 | 1.08 | 0.403 | 0.757 |
| Arginine ^b^ | 104.00 | 64.60 | 0.97 | 0.746 | 0.821 |  | 121.00 | 77.20 | 1.13 | 0.023 | **0.048** | 1.16 | 0.037 | 0.204 |
| Tyrosine ^b^ | 66.60 | 54.70 | 0.92 | 0.727 | 0.821 |  | 77.50 | 50.30 | 1.07 | 0.629 | 0.769 | 1.16 | 0.501 | 0.757 |
| Alanine ^b^ | 475.00 | 229.00 | 1.00 | 0.518 | 0.633 |  | 471.00 | 237.00 | 0.99 | 0.757 | 0.833 | 0.99 | 0.745 | 0.862 |
| Ornithine ^b^ | 111.00 | 96.90 | 1.20 | 0.011 | 0.081 |  | 115.00 | 71.90 | **1.24** | <0.001 | **<0.001** | 1.04 | 0.516 | 0.757 |
| Citrulline ^b^ | 30.20 | 20.30 | 1.13 | 0.059 | **0.152** |  | 30.90 | 18.00 | 1.16 | 0.050 | 0.085 | 1.02 | 0.858 | 0.899 |

^a^ essential amino acids.

^b^ not essential amino acids.

^c^ compared between early-stage and control.

^d^ compared between late-stage and control.

^e^ compared between early-stage and late-stage.

Mann-Whitney U test was used for continuous variables that did not normally distributed.

Benjamini-Hochberg procedure was used for multiple testing correction.
